# Stronger memories through smarter stimulation

**DOI:** 10.7554/eLife.111806

**Published:** 2026-06-09

**Authors:** Justin Riddle

**Affiliations:** 1 Department of Psychology, Florida State University Tallahassee United States

**Keywords:** hippocampus, memories, transcranial magnetic stimulation, hippocampal indirectly targeted stimulation, neurodegenerative diseases, episodic memory, Human

## Abstract

Stimulating brain areas connected to the hippocampus may improve memory function in humans.

**Related research article** Badillo Goicoechea E, Agres PF, Rau JM, San Agustin A, Voss JL. 2026. A meta-analysis suggests that TMS targeting the hippocampal network selectively improves episodic memory. *eLife* **14**:RP108934. doi: 10.7554/eLife.108934.

Episodic memory provides the continuous narrative of our lives from the past to the present. When memory begins to fail, the very fabric of our existence unravels. Disorders of memory, including Alzheimer’s disease, remain difficult to treat effectively. New interventions that can improve memory are therefore desperately needed.

Transcranial magnetic stimulation (TMS) is a non-invasive brain stimulation technique delivered via a coil placed onto the scalp. The TMS coil generates a focused magnetic field that activates neurons in a superficial cortical region. By delivering trains of TMS pulses over days to weeks, TMS can produce lasting changes in brain function. It is now used clinically to treat major depressive disorder and was FDA-approved for this indication in 2008 (Perera et al., 2016).

TMS interventions have since been developed for other psychiatric domains, such as obsessive-compulsive disorder and nicotine-use disorder (Carmi et al., 2019; Dinur-Klein et al., 2014). With its success in psychiatry, many have turned their attention to neurological disorders (Ren et al., 2026). For memory enhancement, the ideal target would be the hippocampus, a structure deep within the medial temporal lobe. However, its location poses a major challenge for direct TMS targeting.

The hippocampus does not act in isolation. Instead, it works in concert with a distributed network of cortical regions (Figure 1). Scientists have addressed the challenge of hippocampal depth by targeting a region of lateral parietal cortex that showed strong functional connectivity with the hippocampus. This approach, known as hippocampal indirectly targeted stimulation, or HITS, improved episodic memory after five consecutive days of stimulation (Wang et al., 2014).

**Figure 1.**
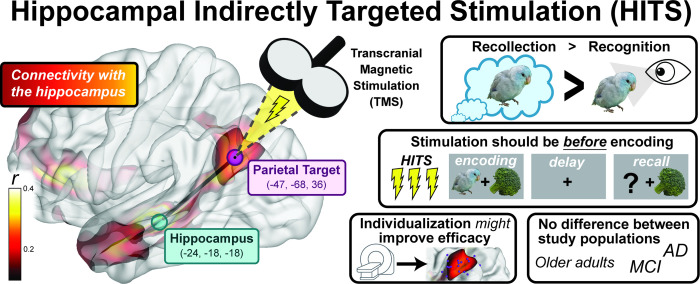
Factors influencing the efficacy of hippocampal indirectly targeted stimulation. Hippocampal indirectly targeted stimulation (HITS) involves delivering transcranial magnetic stimulation (TMS) to a region of the left lateral parietal cortex (magenta) that shows strong functional connectivity with the hippocampus (cyan). The meta-analysis found that memory improvements were greatest when HITS was delivered before encoding and when memory was assessed using recollection-based tasks. Although functional MRI–guided individualized targeting was associated with larger effects, this association did not remain significant after correction for multiple comparisons. HITS was similarly effective in healthy adults, older adults, individuals with mild cognitive impairment, and individuals with Alzheimer’s disease. Coordinates are shown in Montreal Neurological Institute (MNI) standard space, a standardized three-dimensional coordinate system used in brain imaging research to identify locations in the brain consistently across different people. The hippocampal coordinate, derived from previous studies (Wang et al., 2014), served as the seed for connectivity-based targeting, while the parietal coordinate represents the mean stimulation target. Warm colors indicate stronger functional connectivity with the hippocampal seed, as estimated using neurosynth.org, a platform for large-scale, automated synthesis of functional magnetic resonance imaging (fMRI) data.

Since this discovery, many groups replicated these findings or extended them to clinical populations, alternative neural targeting procedures, and different TMS protocols (Freedberg et al., 2022; Jung et al., 2024; Dave et al., 2020; Hermiller et al., 2019). Now, in eLife, Elena Badillo Goicoechea, Phillip Agres, Johanna Rau, Arantzazu San Agustín, and Joel Voss report a meta-analysis of 38 studies that used HITS to improve episodic memory (Badillo Goicoechea et al., 2026).

Their meta-analysis revealed that participants receiving HITS performed better on episodic memory tasks (Hedges’ *g* of 0.44 indicates a small to moderate effect). However, HITS did not improve performance in non-memory tasks. A meta-regression analysis identified three factors that were associated with stronger effects. First, memory tasks that required recollection, such as freely recalling what item was paired with another object, showed greater improvement than tasks that relied primarily on recognition memory, such as judging whether an item had been seen before. Second, HITS was most effective when delivered before encoding new memories. Stimulation delivered during memory consolidation or before retrieval was less effective and, in some cases, even disruptive. When the analysis focused specifically on studies that delivered HITS before encoding and tested memory using recollection-based tasks, the effect size increased to a medium-to-large effect (Hedges’ *g* of 0.66).

A third factor, individualized spatial targeting of the parietal cortex using functional MRI, was initially retained in the model but was not significant after correcting for multiple comparisons. This suggests that personalization of TMS-targeting using MRI may improve efficacy, but more research is needed to determine whether the benefits justify the added cost and complexity.

Intriguingly, the magnitude of memory improvement did not differ across study populations. Older adults and people with mild cognitive impairment or mild-to-moderate Alzheimer’s disease showed benefits comparable to those observed in healthy adults. These findings are promising as researchers begin to investigate the therapeutic potential of HITS.

The broader implication of this work is clear: new treatments are urgently needed for memory decline in healthy aging as well as neurological and psychiatric disorders. However, the studies included in this meta-analysis were primarily basic science studies, and the observed improvements in episodic memory were measured using laboratory-based computerized tasks. A critical next step will be to determine whether improved memory performance in laboratory settings also translates to real-world memory.

Decades of work were required to develop TMS to be used for the treatment of major depression. We may now be at the precipice of a new wave of translational research focused on memory enhancement. If the effects of HITS are strong enough to be clinically meaningful, researchers and clinicians may be able to leverage the existing TMS literature to accelerate this translation.
